# Repository of mutations from Oman: The entry point to a national mutation database

**DOI:** 10.12688/f1000research.6938.1

**Published:** 2015-09-23

**Authors:** Anna Rajab, Nishath Hamza, Salma Al Harasi, Fatma Al Lawati, Una Gibbons, Intesar Al Alawi, Karoline Kobus, Suha Hassan, Ghariba Mahir, Qasim Al Salmi, Barend Mons, Peter Robinson

**Affiliations:** 1 National Genetic Center, Ministry of Health, Muscat, PC 111, Oman; 2 Royal Hospital, Ministry of Health, Muscat, PC 111, Oman; 3 Bio-Semantics at the Department of Medical Informatics, Erasmus Medical Centre, University of Rotterdam, Rotterdam, 3015 CE, Netherlands; 4 Institute of Medical Genetics and Human Genetics, Berlin, 13353, Germany

**Keywords:** Genetic Disease, Birth Defects, disease-associated mutation data, Sultanate of Oman

## Abstract

The Sultanate of Oman is a rapidly developing Muslim country with well-organized government-funded health care services, and expanding medical genetic facilities. The preservation of tribal structures within the Omani population coupled with geographical isolation has produced unique patterns of rare mutations. In order to provide diagnosticians and researchers with access to an up-to-date resource that will assist them in their daily practice we collated and analyzed all of the Mendelian disease-associated mutations identified in the Omani population. By the 1 ^st^ of August 2015, the dataset contained 300 mutations detected in over 150 different genes. More than half of the data collected reflect novel genetic variations that were first described in the Omani population, and most disorders with known mutations are inherited in an autosomal recessive fashion. A number of novel Mendelian disease genes have been discovered in Omani nationals, and the corresponding mutations are included here. The current study provides a comprehensive resource of the mutations in the Omani population published in scientific literature or reported through service provision that will be useful for genetic care in Oman and will be a starting point for variation databases as next-generation sequencing technologies are introduced into genetic medicine in Oman.

## Introduction

**Figure 1.**
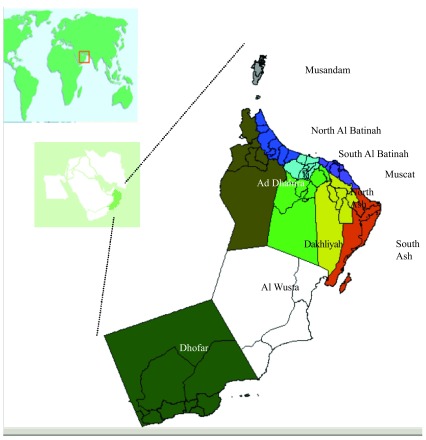
Oman is situated in the South East of the Arabian Peninsula along the East coast of the Arabian Gulf.

Oman is situated in the South East of the Arabian Peninsula along the East coast of the Arabian Gulf ( Figure 1). It has its borders with United Arab Emirates to the North, Saudi Arabia to the West and Yemen to the South West. Oman is the second largest territory in the Arabian Peninsula with an area of 82,000 square miles and a coastline length of 1,300 miles. The native Omani population comprises around 2.2 million inhabitants, and the rate of annual population increase is approximately 25 per 1000. Oman has a young population with nearly half of the population being under 15 years. The Omani population is characterized by a high growth rate, large family size, consanguineous marriages, and the presence of genetic isolates.

Clinical genetic services were introduced in the Sultanate of Oman in the past decade and they have become an important component of health care. This greatly facilitated the systematic collection of data on genetic diseases and birth defects in the past few decades. With the inauguration of the National Genetic Center in 2013, the existing clinical genetic services were supplemented by sophisticated genetic laboratory services.

The amount of published data available on genetic disorders in the Sultanate is considerable. There were a few previous attempts to list the genetic diseases reported in Oman ^1– 4^ and to link them to specific population groups and geographic locations ^5, 6^, analyze population structure ^7^, and to estimate the impact of genetic disorders and birth defects on the community ^4^ and summarize the genetic services available ^8^. The advances in bioinformatics required to annotate human genomic variants and to place them in public data repositories have not kept pace with their discovery. The deposition of such data in the public domain is essential to maximize both their scientific and clinical utility ^9^.

Hence, in the current study we present a comprehensive compilation of germline mutations in nuclear genes associated with human disease in the Omani population.

## Materials and methods

The wealth of genetic variant data in Omani nationals was collected from multiple sources which form a basis for research into genetic conditions reported from Oman. Multiple sources of data were reviewed to form repository of mutations in Omani nationals introduced in this paper. The sources of data included:

(1) 1993–2015 records of patients consulted by clinical geneticists of the Royal Hospital, the largest tertiary hospital in Oman;

(2) 2008–2015 publications curated from PUBMED on birth defects and genetic conditions in Omani nationals. The keywords used were: “Oman”, “Genetic disorders”, “Birth defects”, “mutations”;

(3) 2012–2014 commercial laboratories referral registry at the Royal Hospital for the samples tested overseas.

(4) The internal genetic variant repository of the National Genetic Center << HTTP://ogvd.net>>;

The data presented in this article was manually curated. The OMIM identifiers, Phenotype MIM accession numbers, Phenotype name (OMIM), mutation descriptions, and relevant publications with PMID numbers were all collected from the NCBI database repository. All unavailable through PubMed mutation details were checked with ClinVar, LOVD and CentoMD. The details of unpublished mutations are not included in the present study and feature in Table 2 as “Novel mutations”.

## Results

In this study, a wide range of genetic conditions with known mutations collected in Omani nationals were analysed. The disease classifications are comprised of 44 gene variants causing neurodevelopmental disorders, 21 inborn errors of metabolism, 13 endocrinopathies, 15 skeletal dysplasias, nine disorders of the immune system, four hereditary blood disorders as well as other National groups ( Table 1).

**Table 1.** Range of genetic conditions with known mutations in Omani nationals (details presented in Table 2 and Table 3).

Range of Genetic Conditions with known mutations in Omani nationals	Number of disease causing genes	Number of disease causing mutations	Novel Mutations of known genes	Novel genes and novel genetic mechanisms
Genetic Blood Disorders			
Beta-Globin	1	33	2	3
Alpha-Globin	2	22	3	2
Delta-Globin	1	14	3	0
Other hemolytic and hemorrhagic disorders	3	5	3	2
Neurodevelopmental disorders				
Conditions with intellectual disability	20	23	9	7
Primary Microcephaly	5	5	2	2
Epileptic syndromes	2	2	0	1
Neurodegenerative conditions	6	7	1	1
Syndromic ciliopathies	6	7	5	3
Hereditary spastic paraplegias	5	6	4	3
Neuropathies and neuromuscular disorders	8	13	1	1
Arthrogryposis	3	3	2	2
Inborn errors of metabolism	21	28	21	2
Endocrine disorders	13	28	2	5
Intrahepatic cholestasis and gut anomalies	4	8	3	0
Disorders of the immune system	9	15	3	4
Familial cancers	4	6	1	0
Skeletal dysplasias and osteodysplasias	15	23	6	8
Cardiogenetic conditions	2	2	1	1
Renal disorders and dysplasias	6	13	0	1
Skin, nails, and hair disorders	6	10	0	3
Cutis laxa syndromes	2	7	0	3
Ophthalmological diseases including blindness	5	8	0	2
Congenital deafness	1	3	0	1
Congenital lipodystrophies	5	5	0	1
Cystic fibrosis	1	4	1	2
**Total**	**156**	**300**	**83**	**58**

Extensive genetic studies were performed in Oman for Genetic Blood Disorders and various conditions leading to intellectual disabilities, mental and physical handicap.

In total more than 150 rare genetic disorders were listed in Table 2 and Table 3 with relevant OMIM numbers, PubMed ID (PMID), Gene/Locus name, nucleotide(s) change(s) and the source of the data (PubMed ID Number/ OMIM/ClinVar/LOVD/CentoMD). The names of genetic conditions in Table 2 are stated as found in OMIM “Phenotype-Gene Relationships” table as “Phenotype” arranged in alphabetical order. In Table 3, we present a separate list of 69 known mutations (11–15) that were collected through service provision at the Hemoglobinopathy Laboratory at the National Genetic Center in Oman.

**Table 2.** List of disease-associated mutations in Omani nationals.

No.	Phenotype (OMIM)	OMIM	PMIM ID	Gene/Locus	Nucleotide change	Source: Pubmed ID Nos/ Registration at international databases
1	Achondroplasia	100800	134934	FGFR3	c.749G>C;	LOVD
					c.1172C>A	
2	Adrenal hyperplasia, congenital, due to 21-hydroxylase deficiency	201910	613815	CYP 21A2	p.I236N	21274396
					p.V237E	
					p.M239K	
					c. 306T insert,	
					p.Q318X; conv Cyp 21P to Cyp 21A2	
3	Adrenal hyperplasia, congenital, due to 17-hydroxylase deficiency	202110	609300	CYP17A1	c.287G>A p.Arg96Gln	24498484
4	Allopecia universalis congenita	*203655	602302	HR	c. 2776+1, G>A	9736769
					c.1022T>A	
5	Alport syndrome, autosomal dominant	104199	104200	COL4A3	c.479G>A;	14871398
					c.232delG	
6	Alport syndrome, autosomal recessive	203780	120070	COL4A3	R1215X(CGA>TG	14582039
7	Alport syndrome, X-linked (ATS)	301050	303630	COL 4A5	del exons 7–8 and 31–36	25333070 15149316
					potential duplication of exons 21 – 30+/- exon 20	
8	Alstrom syndrome	*606884	203800	ALMS1	Novel mutation	17594715
9	Amelogenesis imperfecta, type IIA3	*613211	613214	WDR72	c.978T-to-ter	19853237
					(V968X);	
					2857delA	
10	Apparent Mineralocorticoid excess (AME 1);Cortisol 11-beta- ketoreductase deficiency	*218030	614232	HSD11B2	Exon 1: R74G ;	15134813
					Pdelta1nt	
					(P75Delta1nt)	
					Exon2: L114Delta6nt	
					Exon 5: V322ins9nt	
					Exon 3 :A221V	
11	Arterial tortuosity syndrome	208050	208050	SLC2A10	243C-G	16550171
12	Arthrogryposis, renal dysfunction, and cholestasis 1	*608552	208085	Vps33B	c.350delC	15052268
13	Autoimmune Lymphoproliferative Syndrome (Type A)	601859	601853	FAS	c.232 del G, exon3	8787672
14	Bardet-Biedl Syndrome 9 (BBS9)	*615986	615986	PTHB1	IVS 17/IGTA variant	17106446
15	Bardet-Biedl Syndrome 10 (BBS10)	*209900	209900	FLJ23560	n.364fsX368	17106446
					FLJ 23560	
16	Brain Calcifications/Coat’s like syndrome/Rajab syndrome	*613658	613658	NA	Linkage to 2q36.3	19161147
17	Breast cancer	114480	114480	BRCA 1	2080insA	18340530
18	Carbamoylphosphate synthetase I deficiency	*238970	237300	CPSI	c.1590dupT	22106832
19	Carnitinepalmitoyl carboxylase deficiency	*600650	1p32.3	CPT2 gene	detectable mutations were excluded	ClinVar
20	Central hypoventilation syndrome, congenital	603851	209880	PHOX2B	5 Alanine Expansions	ClinVar
					10 Alanine expansions	
21	Ceroid lipofuscinosis, neuronal, 2 (CLN2)	*204500	607998	TPP1	positive linkage	17690061
22	Charcot-Marie-Tooth disease, type 4A	214400	214400	GDAP1	Start-codon mutation	22200116
23	Cohen Syndrome (COH 1)	*216550	216550	VPS13B	7934G>A	15173253
24	Cystic Fibrosis	*219700	7q31.2	CFTR	102T>A+S549R(T>G)]	25829996; 10480369
					delta F508	
					F208Del;	
					S549Rdel	
25	Cholestasis intrahepatic	*243300	243300	ATP8B1	(exon 15) het del	CentoMD; 15239083
26	Cholestasis, benign recurrent intrahepatic, 2	605479	605479	ABCB11	c.149G.A	LOVD; CentoMD
					c.1416T.A	
27	Cholestasis, progressive familial intrahepatic 3	602347	602347	ABCB4	c.2800G>A	LOVD
28	Cholestasis, intrahepatic, of pregnancy, 1	147480	147480	ATP8B1	c.1286A>C;	LOVD; CentoMD
					Novel mutation	
					Novel mutation	
29	Chondrodysplasia, Grebe type (Grebe Acromesomelic Dysplasia)	*200700	200700	GDF5	Del G1144;	16636240
					Transition A1137G	
30	Chronic granulomatous disease due to deficiency of NCF1	*233700	233700	NCF1	c.579G>A	24446915; 24446915
					Novel mutation	
31	Chronic Granulomatous Disease, X-Linked	*306400	306400	CYBB, XK	Del gp91-phox gene (CYBB)	24446915
					del McLeod gene (XK)	
32	Crigler-Naj ar Syndrome, type II	606785	606785	UGT1A1	c.211G>A	9630669
					c.1456T>G	
33	Cutis laxa, autosomal recessive, type IIA (with congenital defect of glycosulation)	*219200	219200	ATP6V0A2	c.294+1G4A	18157129
					c.1929delA	
34	Cutis laxa, autosomal recessive, type IIIB	*614438	614438	PYCR1	356G>A	19648921
					566C>T	
					356G4A	
					c.356G>A	
					c.566C>T	
35	Deafness, autosomal recessive 1A	*220290	2220290	GJB2	S86T	11748849
					35delG	
					167delT
36	Diabetes mellitus, permanent neonatal	606176	606176	ABCC8	c.4480C>T	9769320
37	Dushenne Muscular Dystrophy	320200	320200	DMD	Del exon 7	19449031
					Dupl exons 55 to 77	
					c.4996C>T	
					c.1733_1734delTA	
					c.1175T > G	
					c.1647T>G;	
					c.1521_1523delCTT	
38	Epiphyseal dysplasia, macrocephaly, variable CC agenesis, spindle-shaped fingers, mental retardation	*226900	NA	15q26	Linkage D15S205/ D15S966	11389160
39	Ectodermal dysplasia 1, hypohidrotic, X-linked (EDA)	305100	305100	ED1	c.G1113A; Gly291Arg	11279189
40	Ectodermal dysplasia 10B, hypohidrotic/hair/tooth type, autosomal recessive	224900	224900	EDAR	718delAAA	20979233
41	Enhanced S-cone syndrome (Golden- Favre syndrome)	*268100	268100	NR2E3	c.1117 A>G	24891813
42	Ellis Van-Creveld	*225500	225500	EVC	Frameshift in exon 13	17024374; 20184732
					Novel mutation	
43	Epilepsy, progressive myoclonic 2B (MELF)	254780	254780	NHLRC1	c.468_469delAG	18263761
44	Escobar syndrome	*265000	265000	CHRNG	γ78dup(3)	16826520
45	Ethyl Malonic Aciduria	608451	608451	ETHE1	c.487C>T	Cento MD; 14732903
46	Factor X deficiency/Familiar CRM	*227600	227600	F10	c.381G>A	12574802
47	Fanconi anemia, complementation group D1 (FAD1)	605724	605724	BRCA2	9609C>T	22660720
					exon 25	
48	Fanconi-Bickel Syndrome (GLUT2)	138160	227810	SLC2A2	c.1259G>T;	22660720
					c.1127T>G	
49	Familial Mediterranean fever, AR	*608107	247100	MEFV	c.442G>C	CentoMD, ClinVar
50	Favism	305900	134700	G6PD	c.335A>T	8860013
					G6PD Chatham	
					G6PD A-	
					2 novel mutations	
51	Gastrointestinal defects and immunodeficiency syndrome	243150	243150	TTC7A	Q712X	25534311
52	Geroderma Osteodysplastica Hereditaria	*231070	231070	SCYL1BP1	C-1_1 :GA>CT;	18997784
					257delC	
53	Geroderma osteodysplastica Hereditatria	*231070	231070	GORAB	367G-T	19648921
54	Glaucoma 3A, primary open angle, congenital, juvenile, or adult onset	*231300	231300	CYP1B1	p.G61E	1959767
					p.D374N	
					p.R368H	
					p.E229K	
55	Glycogen Storage Disease II, ACID ALPHA-GLUCOSIDASE DEFICIENCY	232300	232300	GAA	c.2560C>T	ClinVar
					c.2105G>C;	
56	Griscelli syndrome, type 2	607624	607624	RAB27A	Novel mutation	NA
57	Hemolytic uremic syndrome, atypical, susceptibility to, 3	612923	612923	CFI	c.1332A>G	CentoMD
58	Hermansky-Pudlak syndrome 2	608233	608233	AP3B1	c.12_13delTA	16537806 8042664
59	Hyperexplexia	*149400	149400	GLRA1	c.593G>C	22264702
60	Hemophagocytic lymphohistiocytosis, familial, 2	*603553	603553	PRF1	c.265C>A	17674359
					c. 50delT	
					c.265>A	
					.c. 674G>C	
					c.Del12bP	
					c.1122G>A/ Del12bP	
61	Hyperinsulinemic hypoglycemia, familial, 1	*256450	256450	ABCC8	c.4480C>T	9769320; 25972930
					c.96C>A	
					c.563A>G	
					c.119T>G	
					3 novel mutations	
62	Hyperinsulinemic Hypoglycemia, familiar, 5	*147670	609968	INSR	Novel mutation	NA
63	Hypercholesterolemia, familial	*143890	143890	LDLR	c.272delG	23162007; 24249837
64	Hyperlipoproteinemia, type 1D (chylomicronemia)	*615947	615947	GPIHBP1	C.149G>A	22106832
65	HYPERPHENYLALANINEM IA BH4- deficient C, (HPABH4C)	*261630	261630	QDPR	Novel mutation	NA
66	Hyperoxaluria, primary, type 1	259900	259900	AGXT	c.33-34insC	CentoMD; 21612638
67	Homocystinuria due to MTHFR deficiency	*236200	236250	MTHFR	het 677C-T	15053809
68	Hypoparathyroidism-retardation- dysmorphism syndrome (Sanhad- Sakati S)	241410	241410	TBCE	c.155-166del12bp	19491227
69	Hypophosphatasia, childhood	241510	241510	ALPL	c. 98C>T	25023282
70	Huntington Disease	143100	143100	HTT	41-54 repeats	25689972
71	HUNTINTON-LIKE DISEASE	*605613	NIL	HIP1R	Novel mutation	NA
72	Ichthyosis, congenital, autosomal recessive 1	*242300	242300	TGM1	c.278G>A	23689228
					c.396A>H	
73	Insensivity to pain, congenital, with anhydrosis (HSAN IV)	256800	256800	NTRK1	Novel mutation	NA
74	Isovaleric acidemia	*243500	243500	IVD	p.F382fs;	22960500
					p.R392H;	
					p.R395Q;	
					p.E408K	
75	Jouber Syndrome 1 (JBTS1)	*213300	213300	INPPSE	c.1546C>T in exon 7	19668216
76	Joubert syndrome 5	*610142	610188	CEP 290	c.21G>T exone1	19764032
77	Kindler Syndrome (poikiloderma)	*173650	173650	KIND1	R271X	12789646
78	Leprechaunism	147670	246200	INSR	Single nucleotide del in exon 10	OMIM: 147670.0028
79	Leukodystrophy, hypomyelinating, 2 (Pelizaeus-Merzbacher-Like Disease 1)	*608803	608804	GJC2	c.-20+1G>C	23143715
80	LCHAD deficiency	600980	609016	HADHA	Novel mutation	NA
C	Lipodystrophy, congenital generalized, type 4	*613327	613327	PTRF-Cavin	c.160delG	20300641
82	Lipodystrophy, congenital generalized, type 1 (BSCL1)	608594	608594	AGPAT2	Homozygosity D9S1818-DS1826	11916958
83	Lipodystrophy, congenital generalized, type 2 (BSCL2)	606158	269700	SEIPIN	Homozygocity 1883- 4136	12116229,
84	Lipodystrophy, familial partial, 2	150330	151660	LAMIN A\C	Homozygosity 3757	12116229
85	Limb Girdle muscular dystrophy 2B;LGMD2B (Miyoshi myopathy)	254130	254130	DYSF	C :526C>T	10469840
86	Lissencephaly LIS 4A	300121	300121	DCX	exon 5:	11175293
87	Loeys-Dietz syndrome, type 1	609192	609192	TGFBR1 or 2	Positive linkage	16928994
88	Long QT syndrome 1(LQT1)	192500	192500	KCNQ1	1388G>C	15159330
89	MODY type II), Glucokinase related	*125851	125891	GCK	c.757G>T ;	24993573
					c. 292C>T;	
90	Mental retardation, autosomal recessive 43 (MRT 43)	*615817	615817	KIAA1033	c.3056C-G transversion in exon 29	2149877
91	Mental Retardation, autosomal recessive	*602810	602810	HIST 3H3	c. R130C	21937992
92	Mental Retardation Autosomal Recessive, epilepsy, autism	*NA	NA	DEAF1	c.997+4A>C	26048982
93	Meckel Gruber syndrome (MKS 3)	*607361	607361	TMEM67	c. 383-384AC del	16415887
94	Microcephaly with simplified gyral pattern	*603807	603807	NA	Excluded known loci	17975804
95	Microcephaly 3, primary, autosomal recessive	604804	604804	CDK5RAP2	c. E234X	22887808
96	Microcephaly 5, primary, autosomal recessive	608716	608716	ASPM	c.9153_9154 del ins A	15045028
97	Microcephaly and hypomielination	*Omim 179035	NA	PYCR2	c.355C>T	25865492
					c.751C>T	
98	Microcephalic osteodysplastic primordial dwarfism, type II	*210720	210720	PCNT	Maps to 21q22.3	18174396
99	Mucolipidosis IV	252650	252650	MCOLN1	c.1207C>T	15523648; 11030752; 16287144
					Het NM_020533: c.1208G>T.	
100	Mucopolysaccharidosis type IVB (Morquio)	253010	252010	GLB1	c.1420G>C	CentoMD
101	Multiple endocrine neoplasia IIA (MEN2A)	171400	171400	RET	c.1900T>C	8103403
102	Menkes Disease (Kinky Hair Disease)	309400	309400	ATP7A	Novel mutation	CentoMD
				Xq21.1		
103	Multiple pterygium syndrome, lethal type	*100730	253290	CHRNG	c.ARG448TER	16826520
104	Myotonic Dystrophy 1	605377	160900	DMPK	Expansion, >rpts 1allele	8036515
105	Nephrotic syndrome, type 1	*256300	25630	NPHS1	(121delCT) ;	CentoMD; 9660941
					c.218C>T	
106	Nephrotic syndrome, type 2, steroid resistant (NPHS2; SRN1)	*600995	600995	NPHS2	c.467Dup/c.709G>;	CentoMD; 15817495
					c.709G>C	
					c.779T>A;	
107	Noonan syndrome 1 (NS1)	163150	163150	PTPN11	c.218C>T	12161469
108	Niemann-Pick disease, type C1 (NPC1)	*257220	257220	NPC1	c.3362T>G	Cento MD
109	Osteogenesis imperfecta, type VIII	610915	610915	LEPRE1	c.2075-1G>A	24498616; LOVD
					c.1170+9G>A	
110	Osteogenesis imperfecta, type VI	613982	613982	SERPINF1	c.-9+2dup	23054245
111	Osteopetrosis, infantilile malignant	*259700	259700	TCIRG1	c.-XY_-YZdel	23685543
112	Orofaciodigital syndrome V	*174300	174300	DDX59	c.1600G>A	23972372
113	Paroxysmal nonkinesigenic dyskinesia (PNKD1)	*118800	118800	MR-1 gene	c.20C>T : A7V ;	16632198
					c.26C>T : A9V)	
114	Peroxisome biogenesis disorder 1A (Zellweger)	602136	214100	PEX-1	c.1927_1928dupA;. c. 2088A>G	PMCID: 2649967; 25407003
115	Pelger-Huet anomaly	*169400	169400	LBR	del 6 BP in splice site intron 12	21326950
116	Pheochromosytoma/paraganglioma 4	115310	115310	SDHB	c.771dup.A;	25034258; 15328326
					c.574T>C;	
					c.859G>A;	
117	Pituitary hormone deficiency, combined, 3	*221750	221750	LHX3	3,088-bp deletion	18407919
118	Pontocerebellar Hypoplasia type III	*608027	608027	PCLO	nonsense mutation of *PCLO* ( *piccolo*) gene	25832664
119	Polycystic Kidney and Hepatic Disease 1	263200	263200	FCYT	c.107C>T	11919560
120	Polycystic liver disease	608648	608648	SEC63	Del in promoter region	24886261 25165181
121	Rabson-Mendenhall syndrome	262190	262190	INSR	c.671_685dup	CentoMD; 22824672
122	Rajab Syndrome	*613658	613658	NA	linkage D2S351/ D2S2390	19161147
123	Renal tubular acidosis, distal, AR, with hemolitic anaemia	*611590	611590	SLC4A1	A858D;	22126643
					A858D/N	
124	Retinitis pigmentosa-12, autosomal recessive	*604210	600105	RABS 1	7 mutations	24512366
125	Retinitis pigmentosa 37	*604485	268100	NR2E3	p.D406G	24891813
126	Rett Syndrome	312750	312750	MECP2	c.880C>T	ClinVar
127	Robinow syndrome, autosomal recessive	*268310	268310	ROR 2	c.1504C>T	10932186; 19640924
					c.1324C>T	
128	Severe combined immunodeficiency, B cell-negative	179615	601457	RAG1	c.1187G>A	ClinVar
129	Spinal Muscular Atrophy (SMN1)	253300	253300	SMN1	del exons 5, 6, 8 ;	15000810; 17940251
					Del 5q13.2 in exon 7	
130	Spastic paraplegia 18, autosomal recessive (IDMDC)	*611225	611225	ERLIN 2	(D8S1820 and D8S532)	16636240
131	Spastic paraplegia 20, SPG20; (Troyer Syndrome)	*275900	275900	SPG 20	c.123X	20437587
132	Spastic paraplegia 35, autosomal recessive; (FAHN); Leukodystrophy, dysmyelinating, and spastic paraparesis	612319	612319	FA2H	c.235A>C	20104589
133	Spastic paraplegia 54, autosomal recessive	*615033	615003.0005	DDHD2	1546C-T transition	23176823
134	Spastic paraplegia, ataxia, and mental retardation	*607565	607565	GRID2	Novel mutation	NA
135	Split-hand/foot malformation with long bone deficiency 3	*612576	612576	BHLHA9	microduplications	22147889
136	Spondylometaepiphyseal dysplasia, short limb-hand type (SMED-SL)	*271665	271665	DDR2	c.2468_2469del CT	24725993
137	Spondyloepiphyseal dysplasia Omani type with congenital joint dislocations	*143095	1439095	CHST3	c. 911G>A	15215498
138	Spondylocostal dysostosis 2, autosomal recessive	605915	608681	MESP2;	c.880C>T	ClinVar
139	Spinocerebellar ataxia 7; Olivopontocerebellar atrophy III; ADCA type II	164500	164500	ATXN7;	Repeat expansion of ATXN7 gene	ClinVar
140	Schwartz-Jampel syndrome, type 1	*255800	255800	HSPG-2	IVS64DS, A-G, +4; c.1532C>T	11101850
141	Stuve-Wiedemann syndrome/ Schwartz-Jampel type 2 syndrome	601559	601559	LIFR	c.653_654insT	14740318
					c.643del T;	
142	Systemic Lupus Erythematosus (SLE), susceptibility.	*125505	152700	DNASE1L3	G38OR ;	22019780
143	Thanatophoric Dysplasia type 1	*187600	187600	FGFR-3	R248C	12633765
					c.4406A>G	
144	Three-M syndrome 1	*273750	273750	CUL	c2434C>T	19225462
					690 ins C	19877176
145	Three M Syndrome 2	*610991	612921	OBCL1	844ins68	19877176
146	Thrombosis, hyperhomocysteinemic	*236200	236200	CBS	c.807C>A;	16432849
147	Thyroid hormone resistance, autosomal recessive	190160	274300	TRB2	del in exon10	1991834
					Novel mutation	NA
148	von Hippel-Lindau syndrome (VHL)	*193300	193300	VHL	Novel mutation	NA

The disorders are listed in alphabetical order along with the mutations detected in Omani patients. Novel genes and/or mutations identified for the first time in Omani nationals are marked by an asterisk (*). Unpublished mutation data referred as “Novel mutations” would be updated following publication, currently source stated as “NA”.

**Table 3.** Mutations associated with hemoglobin disorders in the Omani population.

Disease	OMIM	Gene/Locus	Nucleotide change	PMID
Sickle cell anemia/ Hemoglobin variants	603903	HBB	c.19G>A	25677748
			c.20A>T	81926
			c.92G>C	25677748
			c.97C>G	1517102
			c.79G>A	25677748
			c.176C>A	25677748
			c.176C>G	25677748
			c.364G>A	25677748
			c.364G>C	25677748
			c.389C>T	25677748
Beta Thalassemia	613985	HBB	c.-151C>T	25677748
			c.-138C>A	25677748
			c.-121C>T*	21801233
			c.-102G>T*	25826385
			c.17_18delCT	25677748
			c.27_28insG	25677748
			c.32C>T	25677748
			c.47G>A	20353347
			c.51delC	20353347
			c.90C>T	25677748
			c.92G>A	25677748
			c.92+1G>A	25677748
			c.92+5G>C	25677748
			c.92+6T>C	25677748
			c.93-22_95del	25677748
			c.93-21G>A	25677748
			c.93-3T>G	25677748
			c.118C>T	25677748
			c.126_129delCTTT	20353347
			c.135delC	25677748
			c.315+1G>A	25677748
			c.316-2A>G	25677748
			c.*108_*112delAATAA	25677748
Alpha Thalassemia	604131	HBA1/HBA2	c.24G>C	25370869
			c.38C>A	25370869
			c.43T>C	25370869
			c.55G>C	24165563
			c.56G>A	25370869
			c.56delG	25370869
			c.64G>C;	25370869
			c.71A>T	5675637
			c.95+2_c.95+6delTGAGG	25370869
			c.118_120delACC	25370869
			c.181A>G	25370869
			c.264C>G	25826385
			c.283_300+3dup*	25370869
			c.326C>A	16840225
			c.427T>C	25370869
			c.*92A>G	25370869
			c.*94A>G	25370869
			Hybrid 3.7 -5 (C>T)*	25370869
			Hybrid 3.7 +46 (C>A)*	25370869
			- -(MED-I); deletion of ~17.5 kb including both alpha-globin genes	25370869
			3.7 kb deletion	25370869
			4.2 kb deletion	25370869
Delta Thalassemia	142000	HBD	c.-118C>T	24985928
			c.14C>T	24985928
			c.49G>C	24985928
			c.68C>A	6058951
			c.82G>T	24985928
			c.93-1G>C	24985928
			c.295G>A	2477064
			c.301C>T	24985928
			c.333-334insGT*	24985928
			c.350G>A	24985928
			c.410G>A	24985938
			c.422C>T	17145605
			c.427G>C*	25043855
			c.443G>T*	24985928

The different mutations reported by the National Genetic Center in patients with Hemoglobinopathies in Oman. Novel mutations are indicated by an asterisk (*) indicated to the left side of the mutation. Mutations are listed in ascending order based on nucleotide position.

For the majority (85%) of rare disorders presented in Table 2, data was derived from publications. The original mutations identified for the first time in Omani population constitute more than half of rare disease data presented in Table 2.

## Discussion

Soon after the completion of the Human Genome Project in 2003, it was clear that the genetic data collected until then presented only a glimpse of the complexity of the human genome and the significance of genetic variants in human disease. Since then, genetic researchers have unearthed innumerable variants that are not only individual-specific; but also ethnicity-, population- and country-specific. Human genetic variation databases have significant implications for both diagnostic and predictive medicine. Often, the pathogenicity of rare mutations is primarily assessed through multiple reports of occurrence in diseased patients that are documented and routinely updated in mutation databases. Given the fact that gene mutations and their frequencies in many Mendelian disorders differ widely between different ethnic groups, even within a country, national databases are highly valuable resources for studies on disease-gene associations, population diversity and genetic history ^10^.

The catalog of Omani mutations presented here will therefore represent a valuable resource that may guide mutation analysis in Omanis suspected of having genetic disease. Unique circumstances in Oman with government-funded comprehensive healthcare throughout the country, and the national coverage for clinical genetics has made the present study possible. Future efforts will be required to extend this database to cover the full spectrum of mutations and population specific variants.

The disease-associated mutation data presented ( Table 1, Table 2, Table 3) show a considerable proportion of novel disease genes as well as novel genetic variants within the Omani population. This was expected due to the presence of inbred and geographically isolated communities, the practice of consanguineous marriages, all of which have tended to skew the allelic spectrum toward rare and private variants within the Omani population. In addition to this, the list of genetic variants also reveals known mutations that were previously reported in certain non-Omani populations, thereby reflecting the historic genetic admixture that occurred in Oman, along the trade routes of a once powerful Omani empire and its foreign colonies. Many of the mutations reported are unique to the Omani population, suggesting a founder effect.

The interest in genetic testing is growing among physicians aiming to provide better medical care and genetic disease prevention. The data collected largely represent mutations of rare autosomal recessively inherited disorders in Oman. The mutation data in Table 2 can be searched by OMIM number, or by disease name. The names of diseases in Table 2 were chosen as described in OMIM in “Phenotype-Gene Relationships” table as “Phenotype” in order to ease finding specific genetic disorders by name.

The number of collected mutations among different disease groups ( Table 1) reflect the frequency of disorders in the Omani population, the burden caused by genetic diseases ^4^, and the interests of individual clinicians in genetic testing.

The knowledge of the genetics of Hemoglobin disorders is among the best in Oman due to national preventive programs and research starting from the 1990s. It is not surprising that around a third of all mutations known in Omani population to date are in four genes causing Hemoglobin disorders ( Table 1, Table 3). The birth prevalence of infants with a hemoglobin disorder was recorded as 3.5–4.7/1,000 ^7, 11^. The frequency of hemoglobin disorders in Oman is among the highest in the world, and may reflect natural selection due to advantage for survival, in the heterozygous state, against malaria. Around 10% of Omani nationals are carriers of the allele for sickle cell anemia, 2–3% carry an allele for Beta-thalassemia and 45% are carriers of an alpha-thalassemia allele ^12– 15^.

Genetic disorders causing disabilities and handicap are of great concern. These are different groups of rare disorders leading to intellectual disability or physical handicap requiring detailed clinical classification, genetic testing, research and preventive measures. The high prevalence of birth defects and genetic conditions in Omani communities causes social, psychological and financial difficulties ^4^. The development and use of national mutation data is of importance to Omani medical care because it not only allows the genetic burden of disease to be quantified, but also provides diagnosticians and researchers access to an up-to-date resource that will assist them in their daily clinical practice and biomedical research ^9^. National databases for genetic variants are also significant from the perspective of preventive healthcare. There is a significant correlation between the occurrence of rare genetic variants associated with Mendelian disease and the burden of morbidity from complex diseases within a population. Heterozygous carriers for recessive disease genes do not manifest the recessive disease but may be at risk of developing complex trait conditions with some similarity in phenotype. For example, heterozygote carriers of mutations in the ataxia telangiectasia gene locus are reportedly susceptible to breast cancer ^16^, and heterozygote carriers of mutations in the glucocerebrosidase ( *GBA*) gene causing Gaucher disease are at an increased risk for Parkinson disease ^17, 18^. Hence, the collection of genetic variant data in national databases will contribute significantly to the prevention of genetic diseases in the population and might greatly impact the management of complex trait diseases in the future. Genetic scientists and international consortiums studying human genetic variation are increasingly interested in dissecting the interplay between genetic makeup and environmental influences on the pattern of diseases worldwide. Current research is expected to create a foundation for the national data online for the benefit of Oman Healthcare.

## Data availability

This article was prepared to introduce the first Omani genetic variation database. This data is available online at HTTP://ogvd.net; raw datasets are not available for Royal Hospital laboratory and clinical data, as the registry contains confidential information that could not be deidentified.
