# Supplementary material for: Repository of mutations from Oman: The entry point to a national mutation database
Source: F1000Res. 2015 Sep 23;4:891. [Version 1] doi: 10.12688/f1000research.6938.1 (PMC4648203; doi:10.12688/f1000research.6938.1)
Supplement: Peer review history

# Peer review: Referee response for version 1

**DOI:** 10.5256/f1000research.7471.r10729

**Published:** 2015-10-14

**Authors:** Shaillay Dogra

**Affiliations:** 1 Vishuo BioMedical Pte Ltd, Singapore, Singapore

Its useful to have region/country specific databases that present a better representation of the prevalence and incidence of region specific diseases and health issues. Putting such data in context of whats known globally shall help in better interpreting whats peculiar to local population and possibly raise some interesting questions on why so? For example, is it the genetic structure or something in the environment?

Some specific comments follow:

1. Authors mention existence of tribal structures, consanguineous marriages etc in Omani society. Is it possible to delineate the effects of these factors on the reported mutations? For example, is this reported in the papers that the authors have collated data from and have the authors noted this aspect in their database?
2. How does the mutation data reported here compare with mutation patterns or frequency seen in other populations? Do the authors provide information on this or link out to other similar resources from other countries? If a physician in Oman is looking up some mutation from this database and wants to know if this is something specific to Oman or is a more general mutation found in other populations too, would this information appear automatically in the database; or could they perform a manual search of the database?
3. Do the authors want to comment about any data privacy issues that maybe associated with such a database, if any and to what extent?
4. Are there any attributes on quality of data in the database? perhaps based on the technique used in the original paper or some other measure some quality metric can be assigned to the mutation information recorded in the database?
5. It would be helpful if authors were able to illustrate out a case or two on how they expect a doctor in Oman to be able to use this database in a real clinical setting; to illustrate the usefulness of the database from a simple collection of data to something that can be used on a more regular basis by doctors in clinical setting.

I have read this submission. I believe that I have an appropriate level of expertise to confirm that it is of an acceptable scientific standard.

# Peer review: Referee response for version 1

**DOI:** 10.5256/f1000research.7471.r10505

**Published:** 2015-10-09

**Authors:** Prajnya Ranganath

**Affiliations:** 1 Department of Medical Genetics, Nizam’s Institute of Medical Sciences, Hyderabad, Telangana, India

1. The paper is well-written and though it does not present any new findings, it can be used as a reference database for Omani mutations.
2. There are a few suggestions for the authors:
  a. Standard HGVS nomenclature should be followed for all the mutations and the authors should preferably stick to one format i.e. either DNA or protein notation. There is no uniformity in the present nomenclature followed in Table 2.
  b. Number of patients in whom each mutation was found, should be indicated – this would give an idea about any preponderance of specific mutations in this population.
  c. Functional validation studies, if available, or at least the mutation prediction scores should be mentioned for the novel sequence variants identified, which will help create a comprehensive database of new likely-pathogenic variants. Again, if the number of patients in whom each of these novel variants were identified is mentioned, we will get an idea as to whether these novel variants were present in more than one case and we can get further proof of the pathogenicity of these mutations.
  d. It would also be interesting to see if there is any ethnic group-wise preponderance of genetic diseases or mutations in the various subsets/ tribes/ regional groups that constitute the Omani population.

I have read this submission. I believe that I have an appropriate level of expertise to confirm that it is of an acceptable scientific standard.
